# Restoration of adrenal function after bilateral adrenal damage due to heparin-induced thrombocytopenia (HIT): a case report

**DOI:** 10.1186/1752-1947-9-18

**Published:** 2015-02-03

**Authors:** Jaafar Jaafar, Françoise Boehlen, Jacques Philippe, Mathieu Nendaz

**Affiliations:** 11 Department of Internal Medicine, Geneva University Hospitals and Faculty of Medicine, Gabrielle-Perret-Gentil 4, Geneva, 1211 Switzerland; 12 Division of Angiology and Hemostasis, Geneva University Hospitals and Faculty of Medicine, Gabrielle-Perret-Gentil 4, Geneva, 1211 Switzerland; 13 Division of Endocrinology and Diabetology, Geneva University Hospitals and Faculty of Medicine, Gabrielle-Perret-Gentil 4, Geneva, 1211 Switzerland

**Keywords:** Adrenal insufficiency, Heparin-induced thrombocytopenia, Adrenal hemorrhage

## Abstract

**Introduction:**

Patients with bilateral adrenal damage due to heparin-induced thrombocytopenia usually need lifelong steroid substitution. So far, no data exists about the natural evolution of such a condition, especially about adrenal function recovery and the real need for lifelong steroids.

**Case presentation:**

An 81-year-old Caucasian woman with bilateral adrenal damage due to heparin-induced thrombocytopenia presented with fever and severe hypotension. Adrenal failure was confirmed biologically and radiologically. She eventually recovered her adrenal function, allowing for steroid withdrawal.

**Conclusions:**

This case report addresses the different mechanisms of adrenal damage due to heparin-induced thrombocytopenia and its natural evolution with potential recovery. This should encourage clinicians to evaluate the real necessity for lifelong corticosteroid substitution in such a condition.

## Introduction

Bilateral adrenal damage (BAD) due to heparin-induced thrombocytopenia (HIT) is increasingly described in the medical literature. It is a devastating condition leading to adrenal insufficiency that needs rapid recognition and subsequent steroid substitution. The main clinical manifestation is hemodynamic collapse. One of the most frequently described mechanisms of adrenal damage is thrombosis of the adrenal veins leading to hemorrhagic necrosis of the glands. In case of adrenal insufficiency, lifelong steroid substitution is generally proposed. However, the natural evolution of such a condition is not well documented [1].

We present the case of a woman with HIT-associated BAD, whose adrenal function recovered, allowing for successful steroids tapering after three months. No recurrence of adrenal insufficiency was observed during two years of follow-up. This case clearly illustrates that recovery of adrenal failure due to HIT-associated BAD is possible. This should incite clinicians to regularly assess adrenal function through basal cortisol dosage and/or an adrenocorticotropic hormone (ACTH) stimulation test. Our report also illustrates different possible mechanisms of adrenal injuries.

## Case presentation

An 81-year-old Caucasian woman with a history of anticoagulated atrial fibrillation (AF), had a sudden cardiac arrest during a hospital visit, requiring 10 minutes of cardiopulmonary resuscitation (CPR) performed immediately after the arrest, allowing the restoration of atrial fibrillation rhythm. She remained intubated for three days. A coronary angiogram showed no significant coronary disease, with a left ventricular ejection fraction of 35%. Pulmonary embolism was ruled out by a computed tomography (CT) scan on day 3, which showed no other anomalies. There was neither electrolyte disturbance nor drugs that could favor such an event. Coronary spasm or idiopathic ventricular fibrillation was finally retained as the cause for her cardiac arrest and an internal defibrillator was implanted. Due to her past AF history (CHADS score = 3), our patient received intravenous anticoagulation with unfractionated heparin (UFH) upon arrival at the intensive care unit (ICU).

**Figure 1:**
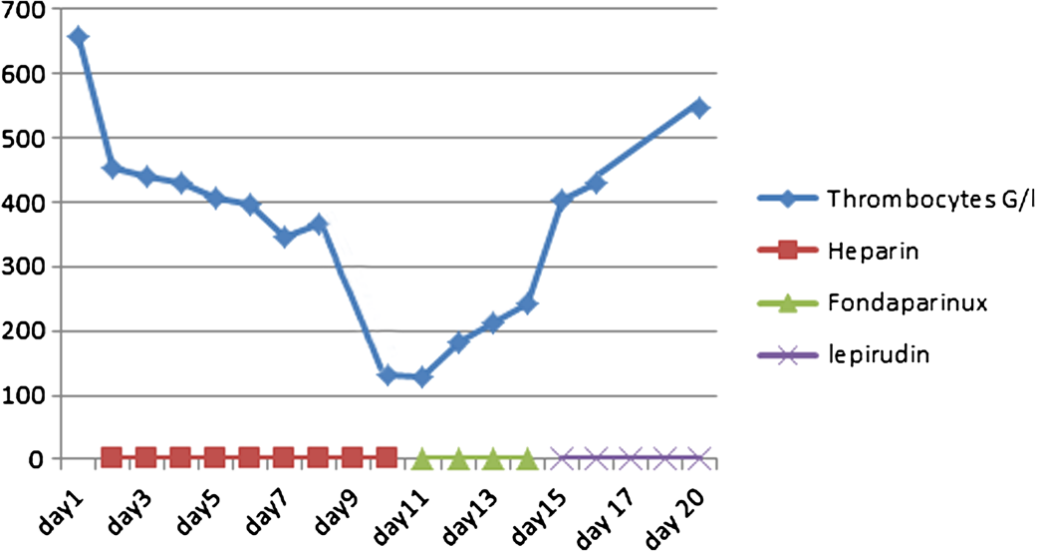
**Platelet count (G/L) during the first 20 days of hospitalization and its relation to heparin use.**

**Figure 2:**
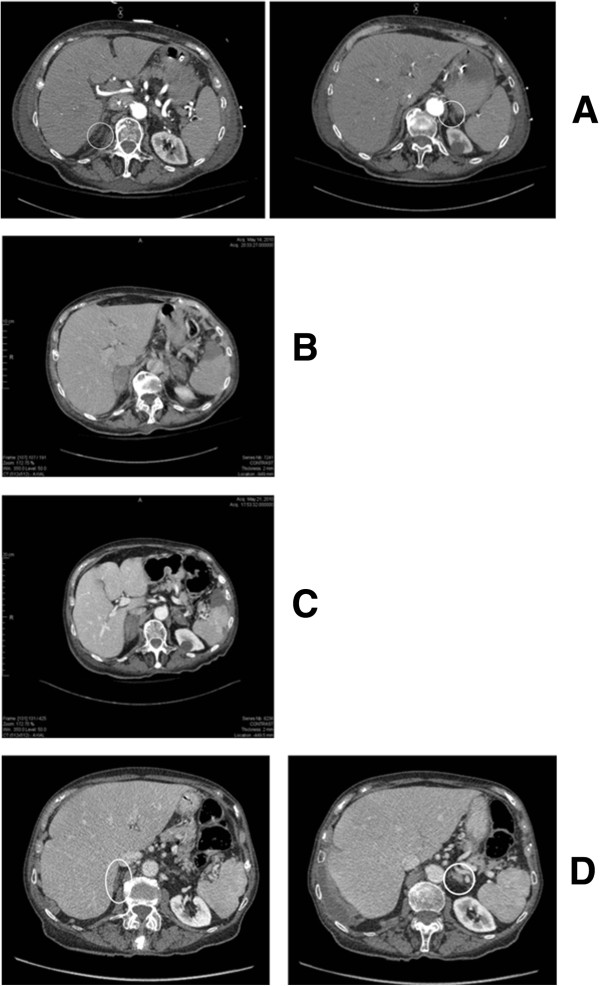
**Abdominal computed tomography scans. (A)** Abdominal computed tomography scan on day 3 showing normal adrenal glands. **(B)** Abdominal computed tomography scan on day 14 showing infiltration with increased adrenal gland volume. **(C)** Abdominal computed tomography scan on day 21 showing bilateral adrenal hemorrhagic infarction. **(D)** Abdominal computed tomography scan on day 435 showing small atrophic adrenal glands.

On day 8, she presented with fever, hypotension and tachycardia. Broad-spectrum antibiotics and inotropic support with dobutamine and noradrenaline were started.Her platelet count dropped from 656 G/L on admission (our patient was known for an essential thrombocytosis Jak-2 positive status) to 131G/L on day 10 and 129G/L on day 11 (Figure 1). Her 4T’s score was 8 and positive anti-PF4-heparin antibodies were confirmed by enzyme-linked immunosorbent assay (ELISA) (day 12). UFH was stopped and therapeutic doses of fondaparinux were immediately started. A thoraco-abdominal CT- scan performed on day 14 showed multiple pulmonary embolisms, splenic infarction and bilateral adrenal ischemia with subsequent hemorrhagic transformation on a CT scan done later on day 21 for non-specific abdominal pain (Figure 2). Diagnosis of HIT with thrombotic complication was retained (HITT) and fondaparinux was switched to lepirudin, at the same time her platelet count was increasing (Figure 1). A Doppler ultrasound (US) scan showed a distal deep venous thrombosis (DVT) of the left peroneal vein (day 17). Her procalcitonin level was lower than 0.5μg/L from day 7 to day 15, with high fibrinogen, which reasonably excluded the diagnosis of sepsis with disseminated intravascular coagulation (DIC). Antiphospholipid syndrome was investigated; anticardiolipin immunoglobulin G (IgG) was slightly positive in this elderly patient, whereas anticardiolipin IgM, anti beta2-glycoprotein antibody IgM and IgG were all negative. Since the thrombotic events happened during heparin therapy and the platelet count increased after heparin cessation, the diagnosis of HITT was maintained.

**Figure 3:**
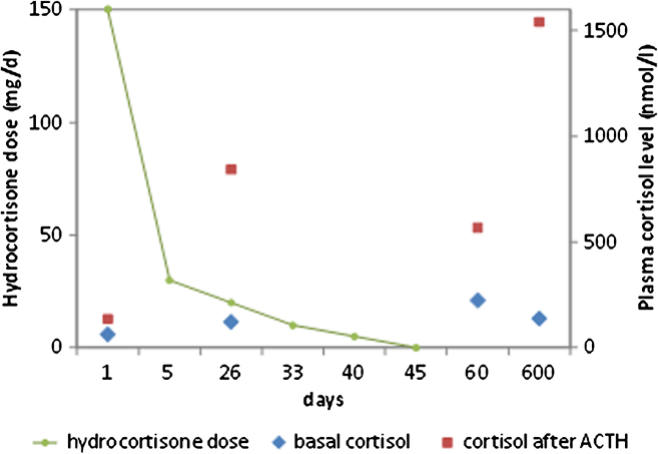
**Hydrocortisone doses and blood cortisol levels before and after adrenocorticotropic hormone tests, over 600 days.**

Basal cortisol on day 15 was 63nmol/L (normal value greater than 500nmol/L) and increased to 136nmol/L 60 minutes after injection of 250μg ACTH (considered normal if the cortisol value exceeds 500nmol/L). The diagnosis of adrenal insufficiency due to bilateral adrenal damage in the context of HITT was made and our patient was given intravenous hydrocortisone 50mg three times a day for five days and then received a maintenance oral dose of 20mg in the morning and 10mg in the afternoon, allowing for a quick hemodynamic improvement. Inotropes and antibiotics were therefore stopped.Following ICU discharge, she was weaned off hydrocortisone progressively over the following weeks (Figure 3), with close blood pressure surveillance, along with plasmatic assessment of potassium and sodium levels twice weekly. After one month of hospitalization, our patient was discharged on 10mg hydrocortisone once daily after a normal ACTH stimulation test. Substitution was tapered further until a complete stop on day 45. Fifteen days after cessation of hydrocortisone (day 60, Figure 3), another ACTH stimulation test without oral substitution showed complete normalization. (Basal cortisol was 224nmol/L and increased to 568nmol/L one hour post-injection of 250μg of tetracosactide.) Two years later, the ACTH test remained normal and showed a basal cortisol at 138nmol/L that increased to 1540nmol/L one hour post-injection of 250μg of tetracosatide (day 600, Figure 3). The patient died of another cause three years after the essential event.

## Discussion

Up to 3 to 5% of patients exposed to UFH for at least five days and 1% of those exposed to low-molecular-weight heparin (LMWH) develop HIT during or after treatment. A subset of these individuals also experience arterial and/or venous thrombosis [2]. About 50% of patients presenting with isolated thrombocytopenia develop a thrombotic event, and approximately 20% of them experience venous thrombosis with DVT and pulmonary embolism (PE) identified as the most common adverse events [3]. Other unusual complications of HIT include adrenal hemorrhage as described in our case [4]. Predictive scores, such as the 4-T score [5], or the HEP score [6], are helpful to assess the likelihood of HIT. Anti-PF4 antibodies disappear in most patients after 90 to 120 days [7]. Treatment consists of discontinuation of heparin and therapeutic anticoagulation with nonheparin antithrombotic agents such as lepirudin and argatroban. LMWH should not be used because of cross-reactivity with HIT antibodies [2]. The safety and efficacy of fondaparinux have not been formally studied in patients with HIT and there is some divergence in its recommendation in this setting [8].

Each adrenal gland is supplied by three suprarenal arteries that originate from the inferior phrenic artery, the renal artery, and the aorta. Their branches feed into a subcapsular plexus located in the adrenal cortex. This highly vascular plexus drains into medullary sinusoids via relatively few venous channels at the corticomedullary junction, thereby creating a potential‘vascular dam’ [9, 10]. Additionally, the adrenal glands are each drained by a single central vein composed of thick longitudinal muscle bundles that create further resistance to blood flow. Any increase in adrenal venous pressure or arterial perfusion pressure may result in hemorrhage into the gland. In cases of hypotension and decreased arterial perfusion, capillaries at the corticomedullary junction are at risk of ischemic necrosis. When normal arterial perfusion is restored, hemorrhage can also occur due to reperfusion injury [10, 11].

High concentrations of catecholamines are present in the adrenal vein during times of stress. This may lead to adrenal vein constriction and increased venous pressure, which can progress to hemorrhage, as described above. Additionally, there is some evidence to suggest that the adrenal vein is prone to development of platelet thrombi in areas of turbulence and local stasis due to catecholamine release [10, 11]. Thrombosis of the adrenal vein has been thought to cause outflow obstruction, leading to adrenal hemorrhage [10, 11].

Heparin provokes adrenal hemorrhage in two ways. First, heparin as an anticoagulant can potentiate bleeding risk when administered during acute illness, thereby leading to adrenal hemorrhage [10]. Second, in the setting of HIT, thrombosis of the central adrenal vein can lead to subsequent adrenal hemorrhage [10]. In a study by Kovacs *et al.* [11], patients exposed to heparin for four to six days and over six days were approximately 17 and 34 times respectively, more likely to develop bilateral massive adrenal hemorrhage than those with no heparin exposure. Patients who developed thrombocytopenia were found to have a 15 times greater risk for hemorrhage.

The presenting signs and symptoms of adrenal failure generally include hypotension or shock, abdominal or flank pain, fever, anorexia, nausea or vomiting, neuropsychiatric symptoms such as confusion or disorientation, and abdominal guarding or rebound tenderness [9]. Laboratory evaluation may be normal or may show a significant alteration of electrolyte levels and a drop in hemoglobin [10]. Traditionally, adrenal insufficiency is diagnosed biochemically by demonstrating low morning cortisol, which is independent from ACTH deficiency and low mineralocorticoid secretion in patients without ACTH deficiency. A short ACTH stimulation test should be performed, unless the diagnosis has been ruled out by a normal basal serum cortisol value [9]. Chronic partial secondary adrenal insufficiency may not be detected by the 250μg ACTH stimulation test. Its use is controversial in critically ill patients, nevertheless there is no other reliable test to assess the adrenocortical axis in this setting [12]. In our case, the adrenal insufficiency diagnosis was highly probable, given the clinical course, the biological tests performed, and the sequence of CT images, although the workup should ideally have included ACTH, renin and aldosterone levels. Adrenal hemorrhage on CT scan is characterized by a round or oval mass in the adrenal gland [13].

When acute adrenal insufficiency is confirmed, immediate treatment involves fluid, electrolyte, and hydrocortisone replacement. Mineralocorticoid replacement can be added as needed once the dose of hydrocortisone is less than 50mg daily [14]. Fludrocortisone, a potent synthetic mineralocorticoid, is given orally at a usual dose of 0.1mg/day. Patients with adrenal insufficiency after bilateral adrenal hemorrhage are usually treated with long-term oral steroids after hospital discharge, with the hypothesis that irreversible adrenal damage has occurred and probably because of lack of sufficient data about its long-term evolution [9, 15]. This raises the question of identifying patients with potential chance of recovery after adrenal damage due to HIT.

To our knowledge, this is the first reported clinical situation demonstrating that some patients can recover their adrenal function within a short period of time after adrenal offense by HIT, allowing for discontinuation of glucocorticoids [16, 17]. For a corticosteroid-weaning trial, one should be able to better characterize the mechanism of adrenal insufficiency and to identify subsets of adrenal damage, including hemorrhagic damage, with partial tissue necrosis. This would ideally be realized by imaging modalities that could show the percentage of residual viable healthy tissue, which could finally indicate the chance of weaning corticosteroids. Until such tests are available to clinicians, our description suggests that a case-by-case evaluation and trial to withdraw steroids under strict medical control may be attempted in adrenal damage due to HIT.

## Conclusions

BAD in the context of HIT is a rare condition that needs urgent recognition, withdrawal of heparin, and immediate substitution treatment with corticosteroids and nonheparin antithrombotic therapy.

Patients usually receive lifelong steroid replacement since the natural evolution of adrenal insufficiency in this context is not known. Our case description suggests that in the context of HIT, the damage mechanism might increase the chance of adrenal recovery, thus allowing for steroid withdrawal during follow-up. A whole gland necrosis might not always be present, especially when venous thrombosis with adrenal congestion is the cause of the deficiency. The next question will be how to identify these patients and which withdrawal protocol would be the most safe and effective.

## Consent

Written informed consent for publication of this case report and accompanying images was unobtainable from the deceased patient's next of kin despite all reasonable efforts. Every effort has been made to protect the identity of our deceased patient and there is no reason to believe that our patient would have objected to publication.

## Abbreviations

ACTH: adrenocorticotropic hormone
AF: atrial fibrillation
BAD: bilateral adrenal damage
CT: computed tomography
CPR: cardiopulmonary resuscitation
DIC: disseminated intravascular coagulation
Doppler US: Doppler ultrasound
DVT: deep venous thrombosis
ELISA: enzyme-linked immunosorbent assay
HIT: heparin-induced thrombocytopenia
HITT: heparin-induced thrombocytopenia/thrombosis
ICU: intensive care unit
IgG: immunoglobulin G
LMWH: low-molecular-weight heparin
PE: pulmonary embolism
UFH: unfractionated heparin.
